# Advanced brain MRI may help understand the link between migraine and multiple sclerosis

**DOI:** 10.1186/s10194-023-01645-7

**Published:** 2023-08-18

**Authors:** Susie Y. Huang, Marc Salomon, Katharina Eikermann-Haerter

**Affiliations:** 1 Athinoula A. Martinos Center for Biomedical Imaging, Department of Radiology, Massachusetts General Hospital, Harvard Medical School, Boston, MA USA; 2 Harvard-MIT Division of Health Sciences and Technology, Massachusetts Institute of Technology, Cambridge, MA USA; 3 Department of Radiology, New York University Langone Medical Center, 660 First Ave, New York, NY 10016 USA

## Abstract

**Background:**

There is a clinical association between migraine and multiple sclerosis.

**Main body:**

Migraine and MS patients share similar demographics, with the highest incidence among young, female and otherwise healthy patients. The same hormonal constellations/changes trigger disease exacerbation in both entities. Migraine prevalence is increased in MS patients, which is further enhanced by disease-modifying treatment. Clinical data show that onset of migraine typically starts years before the clinical diagnosis of MS, suggesting that there is either a unidirectional relationship with migraine predisposing to MS, and/or a “shared factor” underlying both conditions. Brain imaging studies show white matter lesions in both MS and migraine patients. Neuroinflammatory mechanisms likely play a key role, at least as a shared downstream pathway. In this review article, we provide an overview of the literature about 1) the clinical association between migraine and MS as well as 2) brain MRI studies that help us better understand the mechanistic relationship between both diseases with implications on their underlying pathophysiology.

**Conclusion:**

Studies suggest a migraine history predisposes patients to develop MS. Advanced brain MR imaging may shed light on shared and distinct features, while helping us better understand mechanisms underlying both disease entities.

## Background

Migraine is one of the most common neurological disorders, characterized by throbbing/pulsatile unilateral headaches that last for 4–72 h. Thirty percent of migraineurs develop transient neurological symptoms in the setting of an attack, the so-called migraine aura. Aura symptoms characteristically precede or overlap with the headache phase. The most common types of migraine aura involve visual impairment, followed by sensory, language, or motor symptoms [1].

Multiple sclerosis (MS) is the leading non-traumatic cause of neurological disability in young adults, affecting more than 2.2 million individuals globally [2]. MS is characterized by episodes of neurological disability of varying severity and duration, typically on the order of days to weeks in length. Common symptoms include visual loss or double vision and loss of motor function and/or sensation. Progressive disability usually develops over the course of decades and may result in profoundly impaired mobility, cognitive dysfunction and loss of bowel/bladder function. Inflammatory demyelination is considered the hallmark of MS pathology, with axonal degeneration and loss thought to be the substrate of progressive disability [3–7].

Migraine and MS share similar features, see Table 1, as well as comorbidities [8, 9]. Both entities predominantly affect the same demographic group, young and otherwise healthy females. The same environmental factors, for example hormonal constellations during the pre/perimenstrual phase, trigger MS flares and migraine attacks [10]. Neuroinflammation seems to play a key role in both disease entities, at least as a shared downstream pathway. Episodic and chronic courses are possible, and both diseases can cause significant disability. Migraine is the second leading cause of disability in the United States of America, accounting for more than 5% of all disability. About forty percent of patients with MS rely on disability insurance for their income.

**Table 1:** Main findings of selected papers investigating the relationship between migraine and MS

			**Migraine**	**Multiple sclerosis**
**Demographics**	Age at onset		late teens/20 s	30 s
	Gender		F > M (Ratio 2:1)	F > M (Ratio 3:1)
**Incidence**			Lifetime prevalence: 12% of males and 24% of females	Lifetime prevalence: 0.15% of males and 0.45% of females
**Comorbidities**			Multiple Sclerosis, Depression	Migraine, Depression
**Environmental factors**			EBV and other types of infection, stress	EBV and other types of infection, stress
**Clinical symptoms**			Throbbing unilateral headache for 4–72 h, transient neurological impairment	Episodes of neurological disability of varying severity and duration
**Clinical course**			Mostly episodic. Chronic courses can cause significant disability (2nd leading cause of disability in the USA)	Mostly episodic. Chronic courses can cause significant disability (40% of MS patients rely on disability in the USA)
**Brain MRI findings**	Conventional Imaging:		Deep and subcorticalWMHs, increased # with worsening symptoms, dominant side matches HA laterality	Perivenular WMHs (McDonald criteria)
	Advanced Imaging:	Magnetization transfer imaging	Decreased MTR in WMHs	Decreased MTR in WMHs, subsequent increase reflects partial remyelination
		Diffusion tensor imaging	Decreased FA/altered intergrity in optic WM tracts	Increased FA in acute demyelinating lesions; correlates with myelin content and axonal count

There is a clinical association between migraine and MS. In this review article, we summarize evidence for this clinical association, compare shared and disease-specific brain imaging findings as pertinent to better understand the migraine – MS connection, and discuss possible underlying mechanisms.

## Main text

### Evidence for a clinical association between migraine and MS

A clinical association between migraine and MS has been proposed for more than half a century. Already in 1952, Compston described a possible link between migraine and MS, reporting that 2% of MS patients develop migraine within 3 months of MS onset [11]. In 1969, Watkins [12] et al. interviewed 100 consecutive MS clinic patients and 100 random hospital visitors matched for age and sex. The authors found that the incidence of migraine in the MS group was increased with 27% of MS patients reporting migraine compared to 12% in the control group. A later study with blinded design described an increased migraine prevalence in MS patients of 21%, which was higher when compared to the migraine prevalence of 10% in the control group [13]. The strongest evidence to date for an association between migraine and MS comes from a cohort study within the Nurses Health Study II [14]. Migraine status in this study was based on the nurses' report of a physician-diagnosis of migraine. Women who had migraine at enrollment had a 39% increased risk of an incident MS diagnosis over the 15.5 year follow-up period (*p* = 0.008). Another study showed that one third of patients with headaches preceding MS onset had migraine with aura, and that two thirds of those MS patients without a history of headache report the presence of auras [15]. Interestingly, in turn, a diagnosis of MS at baseline was not a risk factor for developing migraine over the follow-up period. A systematic review and meta-analysis by Mirmosayyeb et al. revealed an increased pooled prevalence of migraine in MS patients of 31% [16]. Another systematic review confirmed a significant association between migraine and MS (OR = 2.60) [17]. It should be mentioned that the more recent studies investigating migraine incidence in MS patients may be confounded by disease-modifying treatments, which have been associated with new-onset migraine and worsening of pre-existing migraine [18]. For example, a survey revealed a 46% migraine prevalence in MS patients on interferon treatment [19]. Similarly, a recent online survey revealed a 54% incidence of migraine in MS patients [20]. In summary, most studies report an increased migraine prevalence of 20–45% in MS patients (see Lipton et al. for a review) [19], particularly in patients with relapsing–remitting type MS [21].

The effect of migraine on the clinical course of MS and vice versa has not been well studied yet. However, the NYU MS cohort study showed that MS patients with a history of migraine show more symptoms, pain-related and non-pain related, when compared to those MS patients without migraine [22]. In contrast, presence or absence of migraine in MS patients does not seem to influence the age at MS onset, disease duration or disability [23]. MS patients at first clinical manifestation of their disease showed the highest prevalence of headache, with 78% of MS patients suffering from migraine attacks. Headache prevalence was similarly high in patients with clinically isolated syndrome [24]. MS exacerbation caused a worsening of headaches in two third of MS patients with a history of migraine [15]. In summary, MS patients with headache seem to be younger, have shorter disease duration and are less physically affected than those MS patients without headache. Therefore, headache can be seen as an early MS symptom. In turn, in migraine patients, a history of MS does not seem to affect migraine characteristics such as demographics, clinical presentation and response to therapy. These parameters do not seem to differ between migraine patients with MS and those without [25].

## Neuroimaging in MS and migraine patients

Both a history of migraine and MS predispose to the development of white matter hyperintensities (WMHs). WMHs are non-expansile focal lesions in the deep, subcortical, periventricular or infratentorial white matter [26–30], and thought to be due to gliosis, demyelination and/or loss of axons secondary to inflammatory mechanisms and/or microvascular damage [31]. WMHs are best visualized on T2 and fluid-attenuated inversion recovery (FLAIR) MRI sequences. WMHs might represent a shared end stage of white matter change in patients with migraine and MS, visible with conventional MRI techniques. Advanced MRI techniques investigating subtle changes in WM that appears normal with conventional MRI techniques (“non-affected WM”) may help us better understand the process leading to WMHs in migraine patients, shedding light on differences and similarities between pathophysiology of both disease entities. The process of white matter change has not been fully characterized particularly in migraine patients, and inflammatory mechanisms similar to those seen in MS-related WMHs might be involved.

In patients with migraine, there is a two- to four-fold increased prevalence of WMHs, when compared to controls [26, 32–36]. In contrast to the common age-related WMHs in the general population, migraine is mostly associated with *deep or subcortical* rather than *periventricular* WMHs [29, 37], and cardiovascular risk factors are *not* more prevalent in those migraineurs with WMHs. Interestingly, WMHs in migraineurs seem to occur earlier in life [36], affecting 10% of pediatric migraine patients [38]. WMHs are more commonly seen in patients with migraine with aura than those without aura, and those with a high attack frequency. Another study showed that the number of WMHs increases with intensity of nausea and disability during attacks [39]. Interestingly, progression of WMHs in individuals with migraine was not associated with migraine attack frequency, duration, severity, or anti-migraine treatments [40]. One study reports that the dominant side of WMHs matches the dominant side of headache [41]. Interestingly, some WMHs are only transient, related to migraine attacks. These reversible findings represent regional cerebral vasogenic edema on MRI [42] likely related to vasogenic blood–brain barrier leakage and enhanced permeability of meningeal microvasculature [43].

In MS patients, the dissemination of WMH in space and time has been a key feature of the MS diagnostic criteria, the McDonald criteria [44–47]. Demonstration of WMH in at least two of four locations in the spinal cord and brain (periventricular, juxtacortical, or infratentorial white matter) satisfies the criterion of dissemination in space. Dissemination in time may be satisfied by showing new WMHs in comparison to a baseline reference MRI or simultaneous presence of gadolinium-enhancing and non-enhancing WMHs. WMHs are typically round or ovoid in configuration and tend to follow a perivenular distribution. On FLAIR sequence, MS-typical perivenular T2 hyperintensities are located in the periventricular region and juxtacortical white matter, where blood–brain barrier breakdown takes place. Demyelination along straight medullary venules likely causes the characteristic orientation of MS lesions, perpendicular to the ventricular walls ("Dawson’s fingers”). Interestingly, no difference was found in number or distribution of T2 or enhancing lesion between MS patients with migraine and those without [25]. A recent study showed that a history of migraine in MS patients was associated with a lower hazard ratio of new lesions on MRI [48].

## Advanced MR imaging may help us better understand the migraine—MS association

Advanced MR imaging in patients with MS and migraine helps to further characterize the microstructural substrate of brain changes in both disease entities.

Magnetization transfer imaging is a myelin-sensitive imaging technique, indirectly quantifying the myelin content of white matter [49]. The magnetization transfer ratio (MTR) measures the amount of magnetization exchange between free and macromolecular bound water protons. MTR is affected by demyelination, elevated water content in tissues as a result of inflammation or edema, and/or changes in axonal density [50]. Several studies have suggested the presence of migraine-related focal microstructural damage [51, 52]. The CAMERA-1 and -2 studies showed that normal-appearing white matter that later progressed to WMHs at 9-year follow-up had lower mean MTR at baseline compared to the contralateral white matter. This finding suggests that occult changes in microstructural tissue integrity may precede the development of frank WMHs on conventional T2-weighted MRI [53]. In MS patients, MTR appears decreased in demyelinating lesions, reflecting compromised myelin integrity, although its measurement can be affected by edema, inflammation, and axonal density, reducing its specificity. Dynamic changes in MTR have been measured over time in acute gadolinium-enhancing lesions, with an initial decrease in average lesional MTR followed by an increase that is thought to reflect partial remyelination [54]. In individual lesions, MTR changes correlate with the degree of remyelination and clinical recovery following treatment [43, 55].

Diffusion-weighted imaging uses the Brownian motion of water molecules to characterize tissue microstructure. Diffusion tensor imaging (DTI) models the diffusive motion of water as a tensor and has revealed altered white matter integrity in the corpus callosum [56, 57], optic radiations [58] and corticospinal tracts [59] in patients with migraine. A recent study showed bilateral volume decrease in the occipital white matter adjacent to visual processing cortical areas, not colocalizing with WMHs [60]. Previous DTI studies showed decreased fractional anisotropy (FA) in white matter tracts in the visual processing pathway including the middle temporal region [61] and optic radiations of participants with migraine [58]. Decreased white matter volume makes less myelination due to abnormal maturation or axonal loss a likely explanation [60]. In MS patients, DTI measures have shown some degree of sensitivity and specificity to demyelination and axonal loss. Increased mean diffusivity (MD) and FA appear to reflect demyelination to a greater degree than axonal loss [62, 63]. Radial diffusivity (RD) is also sensitive to myelin content, with increased RD identified in acute demyelinating lesions [64]. RD can differentiate between mild, moderate and severe demyelination but also reflects axonal loss [63]. It has been shown that patients with chronic migraine exhibit widespread increase in RD and MD values in comparison to healthy controls, and decreased FA with increased MD compared to patients with episodic migraine [65]. Advanced diffusion MRI measures incorporating stronger diffusion weighting and multi-compartment models may be more specific to the microstructural changes associated with axonal damage [66, 67]  and may benefit from ultra-high field and high-performance gradient systems that are becoming more widely available [68].

## Possible mechanisms underlying the association of migraine and MS

The nature of the association between migraine and MS is unclear. One of the following two hypotheses to explain the migraine – MS association, or a combination thereof, may be proposed.

First, a unidirectional relationship suggests that migraine predisposes to MS, supported by the clinical observation that migraine typically precedes MS onset by about 7 years [13] and implying that migraine could be a treatable risk factor for MS [15]. Mechanistically, spreading depolarization (SD), the electrophysiologic event underlying migraine and an attack trigger [69], may be a crucial factor for promoting MS onset by facilitating contact between peripheral immune cells and the usually privileged CNS structures. SD increases blood–brain barrier permeability via activating matrix metalloproteinases, thereby initiating neuroinflammation [70]. Elevated levels of MMP-9 and ICAM-1 as well as endothelial cell-specific molecule-1 (ESM-1) and claudin-5 have been observed in migraine patients supporting the involvement of BBB disruption during attacks [71–73]. Therefore, during a migraine attack, circulating immune cells pass the leaky blood brain barrier and may get exposed to myelin antigen in the privileged CNS compartment, causing sensitization. Environmental factors may further trigger the development of autoimmune clones. For example, it has been shown that Ebstein-Barr virus exposure increases the risk of MS [74]. Interestingly, those MS patients with a history of migraine more frequently report exposure to Epstein-Barr virus than do MS patients without a history of migraine [75]. Furthermore, during a migraine attack, SD activates neuronal Pannexin 1 channels that release pro-inflammatory mediators and induce cyclooxygenase-2 / inducible Nitric Oxide synthase expression in astrocytes with microglial activation [76]. Release of cytokines, prostanoids and Nitric Oxide into the subarachnoid space promotes sustained activation of trigeminal nerve fibers surrounding pial vessels, and trigeminal nerve collaterals innervating the middle meningeal artery [77]. In certain cases, a unidirectional relationship between migraine and MS might function in the opposite direction, with an MS lesion in a migraine-relevant pathway initiating migraine. Migraines have been associated with lesions in the brainstem and C2 dorsal horn [78], with the preferential brainstem location of migraine-related lesions being unclear. In particular, migraine onset has been observed with lesion formation in the trigeminocervical complex and periaqueductal gray matter [79, 80]. The trigeminocervical complex is composed of major relay neurons for nociceptive afferent input from the meninges and cervical structures that are important for headache [81] and the periaqueductal gray is an important structure for pain modulation. MS patients with lesions in the periaqueductal gray matter have been shown to display a four-fold increase in migraine-like headaches [82].

Second, increased inflammatory mechanisms might underlie the migraine-MS association [83]. Recent studies suggest that inflammatory mechanisms might also promote the development of WMHs in migraineurs [84], acknowledging that ischemia may be another important underlying mechanism given evidence for increased neuronal vulnerability to ischemia in migraineurs’ brains [85]. There is evidence for a pro-inflammatory baseline state in migraineurs. For example, regulatory T cells that have been shown to suppress mediators of autoimmune responses, the effector T cells, are decreased in migraineurs [86], while peripheral levels of pro-inflammatory cytokines such as IL-1β and TNF-α are increased [87]. Increased peripheral pro-inflammatory cytokines may then activate pain-related CNS structures, as has been shown in animal models. For example, the pro-inflammatory cytokine IL-17A readily crosses the blood–brain-barrier (BBB) and triggers activation of the trigeminovascular complex through microglia-mediated neuroinflammation in a nitroglycerin model of chronic migraine [88]. Furthermore, certain microglial inflammasome, NLRP3, mediate the release of IL-1β and thereby contribute to central sensitization [89]. SD as the electrophysiologic event underlying migraine attacks has been demonstrated to further temporarily upregulate pro-inflammatory cytokines such as IL-6, IL-1β and TNF-α during migraine attacks [90]. A transient increase in the proinflammatory cytokine ICAM-1 and chemokine levels has been confirmed in the jugular blood of migraine patients during attacks [91] and intracranial inflammatory plasma extravasation ipsilateral to the side of headache has been demonstrated with Tc-99 m human serum albumin tracer extravasation in the area of pain [92] as well as gadolinium enhancement close to the middle meningeal artery [93]. Prolonged neuroinflammation during and following migraine attacks has been demonstrated for at least 14 days following a migraine attack by increased glial uptake of the PET TSPO-ligand [11C]PBR28 [94]. Strong persistent extra-axial inflammatory signal was found in the occipital meninges and calvarial bone in migraineurs during and after visual auras, implicating bidirectional crosstalk between brain and skull marrow [95]. In MS pathogenesis and the development of MS-related WMHs, inflammatory mechanisms play a key role, as shown by increased glial uptake of the PET ligand [11C]PBR28, a proxy for neuroinflammation, in both normal appearing white matter and WMHs. Higher levels of microglial activation have been shown to be associated with a greater volume of subsequently enlarging lesions [96], suggesting that innate immune activation contributes to inflammatory neurodegeneration.

## Conclusion

**Fig. 1:**
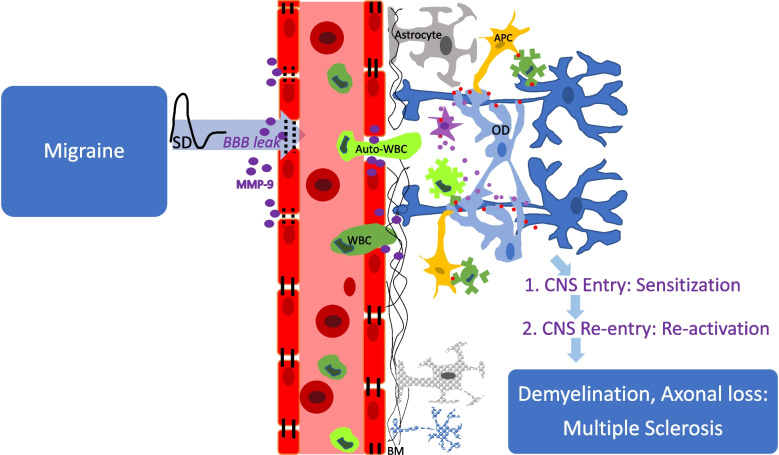
Proposed mechanism on how migraine might facilitate the onset of MS. During a migraine attack, SD causes leakage of the blood–brain-barrier (BBB) (indicated by black dotted lines between endothelial cells) through release of matrix metalloproteinases (MMP-9; purple dots). Peripheral white blood cells (WBC; green) traffic across the permeable BBB. Infiltrated immune cells may get exposed and sensitized to myelin proteins (red dots), via antigen-presenting cells (APC) or through direct exposure from oligodendrocytes (OD). Previously sensitized autoreactive WBC (auto-WBC; light green) may re-enter the CNS from the peripheral circulation during migraine attack-triggered BBB breakdown and release inflammatory mediators (purple dots) with the help of microglia (purple cell), resulting in demyelination and axonal loss.

In summary, there is clinical evidence for an association of migraine and MS. Both clinical studies as well as animal experiments suggest the following scenario to possibly underly the migraine-MS link. Patients with migraine and MS share a pro-inflammatory predisposition. Viral infections or other environmental circumstances trigger the development of T or B autoreactive clones in the peripheral blood. Migraine attacks cause transient opening of the BBB, allowing autoreactive immune cells to enter the CNS. These infiltrated immune cells may get exposed and sensitized to myelin proteins. Previously sensitized autoreactive immune cells may re-enter the CNS from the peripheral circulation during migraine attack-triggered BBB breakdown. These clones may get re-exposed to their respective antigen and release inflammatory mediators with the help of microglia, resulting in demyelination and axonal loss (Fig. 1). Advanced brain MR imaging might shed light on shared and distinct features of migraine and MS, as well as underlying disease mechanisms.

## Data Availability

Not applicable.

## References

[1] Russell MB, Rasmussen BK, Thorvaldsen P, Olesen J (1995). Prevalence and sex-ratio of the subtypes of migraine. Int J Epidemiol.

[2] Collaborators GBDMS (2019) Global, regional, and national burden of multiple sclerosis 1990–2016: a systematic analysis for the Global Burden of Disease Study 2016. Lancet Neurol 18:269–85 10.1016/S1474-4422(18)30443-5 PMC6372756 30679040

[3] Trapp BD, Nave KA (2008). Multiple sclerosis: an immune or neurodegenerative disorder?. Annu Rev Neurosci.

[4] Dutta R, Trapp BD (2011). Mechanisms of neuronal dysfunction and degeneration in multiple sclerosis. Prog Neurobiol.

[5] Stadelmann C (2011). Multiple sclerosis as a neurodegenerative disease: pathology, mechanisms and therapeutic implications. Curr Opin Neurol.

[6] Tallantyre EC, Bo L, Al-Rawashdeh O, Owens T, Polman CH, Lowe JS, Evangelou N (2010). Clinico-pathological evidence that axonal loss underlies disability in progressive multiple sclerosis. Mult Scler.

[7] Ferguson B, Matyszak MK, Esiri MM, Perry VH (1997). Axonal damage in acute multiple sclerosis lesions. Brain.

[8] Vetvik KG, MacGregor EA (2017). Sex differences in the epidemiology, clinical features, and pathophysiology of migraine. Lancet Neurol.

[9] Shahkaram H, Lotfinia S, Mojahed A, Farhangian E, Madadjoo Y (2020). The Rational Emotive Behavioral Group Therapy for Depression and Anger of Patients with Multiple Sclerosis. SN Comprehensive Clin Med.

[10] Roeder HJ, Leira EC (2021). Effects of the Menstrual Cycle on Neurological Disorders. Curr Neurol Neurosci Rep.

[11] Mc AD, Compston N (1952). Some aspects of the natural history of disseminated sclerosis. Q J Med.

[12] Watkins SM, Espir M (1969). Migraine and multiple sclerosis. J Neurol Neurosurg Psychiatry.

[13] Rolak LA, Brown S (1990). Headaches and multiple sclerosis: a clinical study and review of the literature. J Neurol.

[14] Kister I, Munger KL, Herbert J, Ascherio A (2012). Increased risk of multiple sclerosis among women with migraine in the Nurses' Health Study II. Mult Scler.

[15] Tabby D, Majeed MH, Youngman B, Wilcox J (2013). Headache in multiple sclerosis: features and implications for disease management. Int J MS Care.

[16] Mirmosayyeb O, Barzegar M, Nehzat N, Shaygannejad V, Sahraian MA, Ghajarzadeh M (2020). The prevalence of migraine in multiple sclerosis (MS): A systematic review and meta-analysis. J Clin Neurosci.

[17] Pakpoor J, Handel AE, Giovannoni G, Dobson R, Ramagopalan SV (2012). Meta-analysis of the relationship between multiple sclerosis and migraine. PLoS One.

[18] Elmazny A, Hamdy SM, Abdel-Naseer M, Shalaby NM, Shehata HS, Kishk NA, Nada MA, Mourad HS, Hegazy MI, Abdelalim A, Ahmed SM, Hatem G, Fouad AM, Mahmoud H, Hassan A (2020). Interferon-beta-induced headache in patients with multiple sclerosis: frequency and characterization. J Pain Res.

[19] Kister I, Caminero AB, Herbert J, Lipton RB (2010). Tension-type headache and migraine in multiple sclerosis. Curr Pain Headache Rep.

[20] Fragoso YD, Adoni T, Alves-Leon SV, Apostolos-Pereira SL, Carneiro MAD, Chikota EM, Diniz DS, Eboni ACB, Gomes S, Goncalves MVM, Goncalves RP, Inojosa JL, Junqueira TF, Machado SC, Malfetano FR, Mansur LF, Mendes MF, Muniz A, Nobrega Junior AW, Olival GSD, Parolin MF, Pimentel MLV, Rocha CF, Ruocco HH, Santos GC, Siquineli F, Soares JOD, Sousa NAC, Tauil CB, Winckler TCA (2019). Migraine in 746 patients with multiple sclerosis. Arq Neuropsiquiatr.

[21] Nicoletti A, Patti F, Lo Fermo S, Liberto A, Castiglione A, Laisa P, Garifoli A, La Naia F, Maimone D, Sorbello V, Contrafatto D, Zappia M (2008). Headache and multiple sclerosis: a population-based case-control study in Catania. Sicily Cephalalgia.

[22] Burstein R, Yarnitsky D, Goor-Aryeh I, Ransil BJ, Bajwa ZH (2000). An association between migraine and cutaneous allodynia. Ann Neurol.

[23] D'Amico D, La Mantia L, Rigamonti A, Usai S, Mascoli N, Milanese C, Bussone G (2004). Prevalence of primary headaches in people with multiple sclerosis. Cephalalgia.

[24] Gebhardt M, Kropp P, Hoffmann F, Zettl UK (2019). Headache in the course of multiple sclerosis: a prospective study. J Neural Transm (Vienna).

[25] Kister I, Caminero AB, Monteith TS, Soliman A, Bacon TE, Bacon JH, Kalina JT, Inglese M, Herbert J, Lipton RB (2010). Migraine is comorbid with multiple sclerosis and associated with a more symptomatic MS course. J Headache Pain.

[26] Bashir A, Lipton RB, Ashina S, Ashina M (2013). Migraine and structural changes in the brain: a systematic review and meta-analysis. Neurology.

[27] Palm-Meinders IH, Koppen H, Terwindt GM, Launer LJ, Konishi J, Moonen JME, Bakkers JTN, Hofman PAM, van Lew B, Middelkoop HAM, van Buchem MA, Ferrari MD, Kruit MC (2012). Structural brain changes in migraine. JAMA.

[28] Kurth T, Mohamed S, Maillard P, Zhu Y-C, Chabriat H, Mazoyer B, Bousser M-G, Dufouil C, Tzourio C (2011). Headache, migraine, and structural brain lesions and function: population based Epidemiology of Vascular Ageing-MRI study. BMJ.

[29] Kruit MC, van Buchem MA, Hofman PA, Bakkers JT, Terwindt GM, Ferrari MD, Launer LJ (2004). Migraine as a risk factor for subclinical brain lesions. JAMA.

[30] Kruit MC, Launer LJ, Ferrari MD, van Buchem MA (2006). Brain stem and cerebellar hyperintense lesions in migraine. Stroke.

[31] Porter A, Gladstone JP, Dodick DW (2005). Migraine and white matter hyperintensities. Curr Pain Headache Rep.

[32] Kruit MC, Launer LJ, Ferrari MD, van Buchem MA (2005). Infarcts in the posterior circulation territory in migraine The population-based MRI CAMERA study. Brain.

[33] Swartz RH, Kern RZ (2004). Migraine is associated with magnetic resonance imaging white matter abnormalities: a meta-analysis. Arch Neurol.

[34] Zhang Q, Datta R, Detre JA, Cucchiara B (2017). White matter lesion burden in migraine with aura may be associated with reduced cerebral blood flow. Cephalalgia.

[35] Gaist D, Garde E, Blaabjerg M, Nielsen HH, Krøigård T, Østergaard K, Møller HS, Hjelmborg J, Madsen CG, Iversen P, Kyvik KO, Siebner HR, Ashina M (2016). Migraine with aura and risk of silent brain infarcts and white matter hyperintensities: an MRI study. Brain.

[36] Hamedani AG, Rose KM, Peterlin BL, Mosley TH, Coker LH, Jack CR, Knopman DS, Alonso A, Gottesman RF (2013). Migraine and white matter hyperintensities: the ARIC MRI study. Neurology.

[37] Kurth T, Mohamed S, Maillard P, Zhu YC, Chabriat H, Mazoyer B, Bousser MG, Dufouil C, Tzourio C (2011). Headache, migraine, and structural brain lesions and function: population based Epidemiology of Vascular Ageing-MRI study. BMJ.

[38] Eidlitz-Markus T, Zeharia A, Haimi-Cohen Y, Konen O (2013). MRI white matter lesions in pediatric migraine. Cephalalgia.

[39] Negm M, Housseini AM, Abdelfatah M, Asran A (2018). Relation between migraine pattern and white matter hyperintensities in brain magnetic resonance imaging. Egypt J Neurol Psychiatr Neurosurg.

[40] Kruit MC, van Buchem MA, Launer LJ, Terwindt GM, Ferrari MD (2010). Migraine is associated with an increased risk of deep white matter lesions, subclinical posterior circulation infarcts and brain iron accumulation: the population-based MRI CAMERA study. Cephalalgia.

[41] Del Sette M, Dinia L, Bonzano L, Roccatagliata L, Finocchi C, Parodi RC, Sivori G, Gandolfo C (2008). White matter lesions in migraine and right-to-left shunt: a conventional and diffusion MRI study. Cephalalgia.

[42] Resnick S, Reyes-Iglesias Y, Carreras R, Villalobos E (2006). Migraine with aura associated with reversible MRI abnormalities. Neurology.

[43] Chen JT, Collins DL, Atkins HL, Freedman MS, Arnold DL (2008). Canadian MSBMTSG: Magnetization transfer ratio evolution with demyelination and remyelination in multiple sclerosis lesions. Ann Neurol.

[44] McDonald WI, Compston A, Edan G, Goodkin D, Hartung HP, Lublin FD, McFarland HF, Paty DW, Polman CH, Reingold SC, Sandberg-Wollheim M, Sibley W, Thompson A, van den Noort S, Weinshenker BY, Wolinsky JS (2001). Recommended diagnostic criteria for multiple sclerosis: guidelines from the International Panel on the diagnosis of multiple sclerosis. Ann Neurol.

[45] Polman CH, Reingold SC, Banwell B, Clanet M, Cohen JA, Filippi M, Fujihara K, Havrdova E, Hutchinson M, Kappos L, Lublin FD, Montalban X, O'Connor P, Sandberg-Wollheim M, Thompson AJ, Waubant E, Weinshenker B, Wolinsky JS (2011). Diagnostic criteria for multiple sclerosis: 2010 revisions to the McDonald criteria. Ann Neurol.

[46] Polman CH, Reingold SC, Edan G, Filippi M, Hartung HP, Kappos L, Lublin FD, Metz LM, McFarland HF, O'Connor PW, Sandberg-Wollheim M, Thompson AJ, Weinshenker BG, Wolinsky JS (2005). Diagnostic criteria for multiple sclerosis: 2005 revisions to the "McDonald Criteria". Ann Neurol.

[47] Thompson AJ, Banwell BL, Barkhof F, Carroll WM, Coetzee T, Comi G, Correale J, Fazekas F, Filippi M, Freedman MS, Fujihara K, Galetta SL, Hartung HP, Kappos L, Lublin FD, Marrie RA, Miller AE, Miller DH, Montalban X, Mowry EM, Sorensen PS, Tintore M, Traboulsee AL, Trojano M, Uitdehaag BMJ, Vukusic S, Waubant E, Weinshenker BG, Reingold SC, Cohen JA (2018). Diagnosis of multiple sclerosis: 2017 revisions of the McDonald criteria. Lancet Neurol.

[48] Salter A, Kowalec K, Fitzgerald KC, Cutter G, Marrie RA (2020). Comorbidity is associated with disease activity in MS: Findings from the CombiRx trial. Neurology.

[49] Sled JG, Pike GB (2001). Quantitative imaging of magnetization transfer exchange and relaxation properties in vivo using MRI. Magn Reson Med.

[50] Schmierer K, Scaravilli F, Altmann DR, Barker GJ, Miller DH (2004). Magnetization transfer ratio and myelin in postmortem multiple sclerosis brain. Ann Neurol.

[51] Granziera C, Daducci A, Romascano D, Roche A, Helms G, Krueger G, Hadjikhani N (2014). Structural abnormalities in the thalamus of migraineurs with aura: a multiparametric study at 3 T. Hum Brain Mapp.

[52] Granziera C, Romascano D, Daducci A, Roche A, Vincent M, Krueger G, Hadjikhani N (2013). Migraineurs without aura show microstructural abnormalities in the cerebellum and frontal lobe. Cerebellum.

[53] Arkink EB, Palm-Meinders IH, Koppen H, Milles J, van Lew B, Launer LJ, Hofman PAM, Terwindt GM, van Buchem MA, Ferrari MD, Kruit MC (2019). Microstructural white matter changes preceding white matter hyperintensities in migraine. Neurology.

[54] Giacomini PS, Levesque IR, Ribeiro L, Narayanan S, Francis SJ, Pike GB, Arnold DL (2009). Measuring demyelination and remyelination in acute multiple sclerosis lesion voxels. Arch Neurol.

[55] Brown RA, Narayanan S, Stikov N, Cook S, Cadavid D, Wolansky L, Arnold DL (2016). MTR recovery in brain lesions in the BECOME study of glatiramer acetate vs interferon beta-1b. Neurology.

[56] Yu D, Yuan K, Zhao L, Dong M, Liu P, Yang X, Liu J, Sun J, Zhou G, Xue T, Zhao L, Cheng P, Dong T, von Deneen KM, Qin W, Tian J (2013). White matter integrity affected by depressive symptoms in migraine without aura: a tract-based spatial statistics study. NMR Biomed.

[57] Yuan K, Qin W, Liu P, Zhao L, Yu D, Zhao L, Dong M, Liu J, Yang X, von Deneen KM, Liang F, Tian J (2012). Reduced fractional anisotropy of corpus callosum modulates inter-hemispheric resting state functional connectivity in migraine patients without aura. PLoS One.

[58] Rocca MA, Pagani E, Colombo B, Tortorella P, Falini A, Comi G, Filippi M (2008). Selective diffusion changes of the visual pathways in patients with migraine: a 3-T tractography study. Cephalalgia.

[59] Chong CD, Schwedt TJ (2015). Migraine affects white-matter tract integrity: A diffusion-tensor imaging study. Cephalalgia.

[60] Palm-Meinders IH, Arkink EB, Koppen H, Amlal S, Terwindt GM, Launer LJ, van Buchem MA, Ferrari MD, Kruit MC (2017). Volumetric brain changes in migraineurs from the general population. Neurology.

[61] Granziera C, DaSilva AF, Snyder J, Tuch DS, Hadjikhani N (2006). Anatomical alterations of the visual motion processing network in migraine with and without aura. PLoS Med.

[62] Schmierer K, Wheeler-Kingshott CA, Boulby PA, Scaravilli F, Altmann DR, Barker GJ, Tofts PS, Miller DH (2007). Diffusion tensor imaging of post mortem multiple sclerosis brain. Neuroimage.

[63] Klawiter EC, Schmidt RE, Trinkaus K, Liang HF, Budde MD, Naismith RT, Song SK, Cross AH, Benzinger TL (2011). Radial diffusivity predicts demyelination in ex vivo multiple sclerosis spinal cords. Neuroimage.

[64] Fox RJ, Cronin T, Lin J, Wang X, Sakaie K, Ontaneda D, Mahmoud SY, Lowe MJ, Phillips MD (2011). Measuring myelin repair and axonal loss with diffusion tensor imaging. AJNR Am J Neuroradiol.

[65] Coppola G, Di Renzo A, Tinelli E, Petolicchio B, Di Lorenzo C, Parisi V, Serrao M, Calistri V, Tardioli S, Cartocci G, Caramia F, Di Piero V, Pierelli F (2020). Patients with chronic migraine without history of medication overuse are characterized by a peculiar white matter fiber bundle profile. J Headache Pain.

[66] Huang SY, Fan Q, Machado N, Eloyan A, Bireley JD, Russo AW, Tobyne SM, Patel KR, Brewer K, Rapaport SF, Nummenmaa A, Witzel T, Sherman JC, Wald LL, Klawiter EC (2019). Corpus callosum axon diameter relates to cognitive impairment in multiple sclerosis. Ann Clin Transl Neurol.

[67] Huang SY, Tobyne SM, Nummenmaa A, Witzel T, Wald LL, McNab JA, Klawiter EC (2016). Characterization of Axonal Disease in Patients with Multiple Sclerosis Using High-Gradient-Diffusion MR Imaging. Radiology.

[68] Vachha B, Huang SY (2021) MRI with ultrahigh field strength and high-performance gradients: challenges and opportunities for clinical neuroimaging at 7 T and beyond Abstract European Radiology Experimental 5(1). 10.1186/s41747-021-00216-2 10.1186/s41747-021-00216-2 PMC8387544 34435246

[69] Eikermann-Haerter K, Dilekoz E, Kudo C, Savitz SI, Waeber C, Baum MJ, Ferrari MD, van den Maagdenberg AM, Moskowitz MA, Ayata C (2009). Genetic and hormonal factors modulate spreading depression and transient hemiparesis in mouse models of familial hemiplegic migraine type 1. J Clin Invest.

[70] Gursoy-Ozdemir Y, Qiu J, Matsuoka N, Bolay H, Bermpohl D, Jin H, Wang X, Rosenberg GA, Lo EH, Moskowitz MA (2004). Cortical spreading depression activates and upregulates MMP-9. J Clin Invest.

[71] Imamura K, Takeshima T, Fusayasu E, Nakashima K (2008). Increased plasma matrix metalloproteinase-9 levels in migraineurs. Headache.

[72] Wang F, He Q, Ren Z, Li F, Chen W, Lin X, Zhang H, Tai G (2015). Association of serum levels of intercellular adhesion molecule-1 and interleukin-6 with migraine. Neurol Sci.

[73] Yucel M, Kotan D, Gurol Ciftci G, Ciftci IH, Cikriklar HI (2016). Serum levels of endocan, claudin-5 and cytokines in migraine. Eur Rev Med Pharmacol Sci.

[74] Bjornevik K, Cortese M, Healy BC, Kuhle J, Mina MJ, Leng Y, Elledge SJ, Niebuhr DW, Scher AI, Munger KL, Ascherio A (2022). Longitudinal analysis reveals high prevalence of Epstein-Barr virus associated with multiple sclerosis. Science.

[75] Ascherio A, Munger KL (2007). Environmental risk factors for multiple sclerosis Part II: Noninfectious factors. Ann Neurol.

[76] Chen SP, Qin T, Seidel JL, Zheng Y, Eikermann M, Ferrari MD, van den Maagdenberg A, Moskowitz MA, Ayata C, Eikermann-Haerter K (2017). Inhibition of the P2X7-PANX1 complex suppresses spreading depolarization and neuroinflammation. Brain.

[77] Karatas H, Erdener SE, Gursoy-Ozdemir Y, Lule S, Eren-Kocak E, Sen ZD, Dalkara T (2013). Spreading depression triggers headache by activating neuronal Panx1 channels. Science.

[78] Putzki N, Katsarava Z (2010). Headache in multiple sclerosis. Curr Pain Headache Rep.

[79] Haas DC, Kent PF, Friedman DI (1993). Headache caused by a single lesion of multiple sclerosis in the periaqueductal gray area. Headache.

[80] Fragoso YD, Brooks JB (2007). Two cases of lesions in brainstem in multiple sclerosis and refractory migraine. Headache.

[81] Bartsch T, Goadsby PJ (2003). The trigeminocervical complex and migraine: current concepts and synthesis. Curr Pain Headache Rep.

[82] Gee JR, Chang J, Dublin AB, Vijayan N (2005). The association of brainstem lesions with migraine-like headache: an imaging study of multiple sclerosis. Headache.

[83] Gelfand AA, Gelfand JM, Goadsby PJ (2013). Migraine and multiple sclerosis: Epidemiology and approach to treatment. Mult Scler Relat Disord.

[84] Eikermann-Haerter K, Huang SY (2021). White matter lesions in migraine. Am J Pathol.

[85] Eikermann-Haerter K (2021). Neuronal plumes initiate spreading depolarization, the electrophysiologic event driving migraine and stroke. Neuron.

[86] Faraji F, Shojapour M, Farahani I, Ganji A, Mosayebi G (2021). Reduced regulatory T lymphocytes in migraine patients. Neurol Res.

[87] Biscetti L, De Vanna G, Cresta E, Corbelli I, Gaetani L, Cupini L, Calabresi P, Sarchielli P (2021). Headache and immunological/autoimmune disorders: a comprehensive review of available epidemiological evidence with insights on potential underlying mechanisms. J Neuroinflammation.

[88] Chen H, Tang X, Li J, Hu B, Yang W, Zhan M, Ma T, Xu S (2022). IL-17 crosses the blood-brain barrier to trigger neuroinflammation: a novel mechanism in nitroglycerin-induced chronic migraine. J Headache Pain.

[89] He W, Long T, Pan Q, Zhang S, Zhang Y, Zhang D, Qin G, Chen L, Zhou J (2019). Microglial NLRP3 inflammasome activation mediates IL-1beta release and contributes to central sensitization in a recurrent nitroglycerin-induced migraine model. J Neuroinflammation.

[90] Torun E, Kahraman FU, Goksu AZ, Vahapoglu A, Cakin ZE (2019). Serum catalase, thiol and myeloperoxidase levels in children passively exposed to cigarette smoke. Ital J Pediatr.

[91] Sarchielli P, Alberti A, Baldi A, Coppola F, Rossi C, Pierguidi L, Floridi A, Calabresi P (2006). Proinflammatory cytokines, adhesion molecules, and lymphocyte integrin expression in the internal jugular blood of migraine patients without aura assessed ictally. Headache.

[92] Knotkova H, Pappagallo M (2007). Imaging intracranial plasma extravasation in a migraine patient: a case report. Pain Med.

[93] Arnold G, Reuter U, Kinze S, Wolf T, Einhaupl KM (1998). Migraine with aura shows gadolinium enhancement which is reversed following prophylactic treatment. Cephalalgia.

[94] Albrecht DS, Mainero C, Ichijo E, Ward N, Granziera C, Zurcher NR, Akeju O, Bonnier G, Price J, Hooker JM, Napadow V, Loggia ML, Hadjikhani N (2019). Imaging of neuroinflammation in migraine with aura: A [(11)C]PBR28 PET/MRI study. Neurology.

[95] Hadjikhani N, Albrecht DS, Mainero C, Ichijo E, Ward N, Granziera C, Zurcher NR, Akeju O, Bonnier G, Price J, Hooker JM, Napadow V, Nahrendorf M, Loggia ML, Moskowitz MA (2020). Extra-axial inflammatory signal in parameninges in migraine with visual aura. Ann Neurol.

[96] Datta G, Colasanti A, Rabiner EA, Gunn RN, Malik O, Ciccarelli O, Nicholas R, Van Vlierberghe E, Van Hecke W, Searle G, Santos-Ribeiro A, Matthews PM (2017). Neuroinflammation and its relationship to changes in brain volume and white matter lesions in multiple sclerosis. Brain.

